# Supplementary material for: Inflammatory factors and risk of lung adenocarcinoma: a Mendelian randomization study mediated by blood metabolites
Source: Front Endocrinol (Lausanne). 2024 Aug 27;15:1446863. doi: 10.3389/fendo.2024.1446863 (PMC11384989; doi:10.3389/fendo.2024.1446863)
Supplement: Supplementary file 1 [file DataSheet1.zip › supplementary files/S2/GCST90200392/forest.pdf]

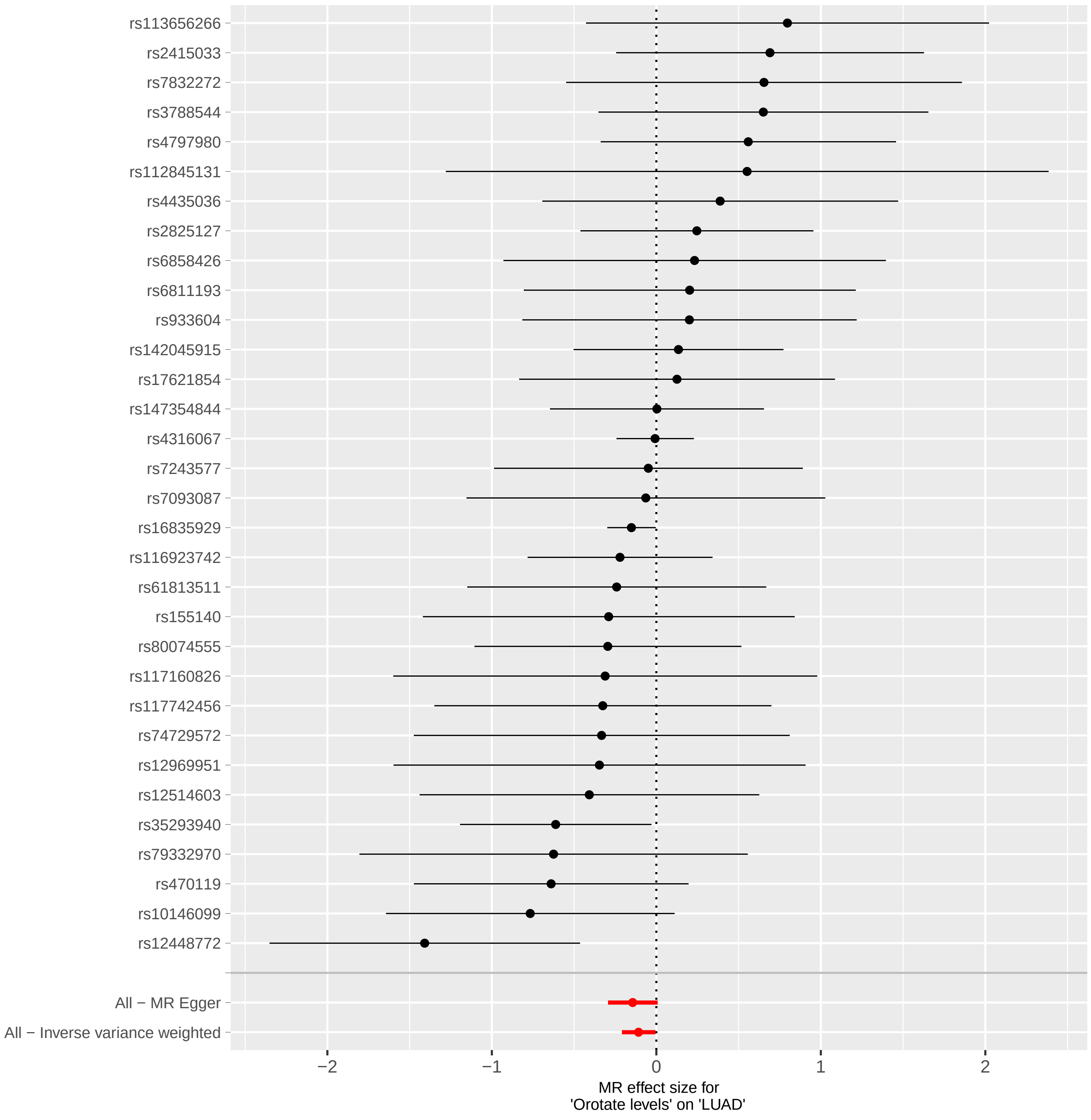
rs113656266
rs2415033
rs7832272
rs3788544
rs4797980
rs112845131
rs4435036
rs2825127
rs6858426
rs6811193
rs933604
rs142045915
rs17621854
rs147354844
rs4316067
rs7243577
rs7093087
rs16835929
rs116923742
rs61813511
rs155140
rs80074555
rs117160826
rs117742456
rs74729572
rs12969951
rs12514603
rs35293940
rs79332970
rs470119
rs10146099
rs12448772
All − MR Egger
All − Inverse variance weighted
−2
−1
0
1
2
MR effect size for
'Orotate levels' on 'LUAD'
